# Carbide Dihydrides: Carbonaceous Species Identified in Ta_4_^+^‐Mediated Methane Dehydrogenation

**DOI:** 10.1002/anie.202010794

**Published:** 2020-10-22

**Authors:** Jozef Lengyel, Nikita Levin, Frank J. Wensink, Olga V. Lushchikova, Robert N. Barnett, Uzi Landman, Ueli Heiz, Joost M. Bakker, Martin Tschurl

**Affiliations:** ^1^ Lehrstuhl für Physikalische Chemie Technische Universität München Lichtenbergstraße 4 85748 Garching Germany; ^2^ Radboud University Institute for Molecules and Materials FELIX Laboratory Toernooiveld 7 6525 ED Nijmegen The Netherlands; ^3^ School of Physics Georgia Institute of Technology Atlanta GA 30332 USA

**Keywords:** bond activation, infrared spectroscopy, methane conversion, DFT calculations, tantalum cluster

## Abstract

The products of methane dehydrogenation by gas‐phase Ta_4_^+^ clusters are structurally characterized using infrared multiple photon dissociation (IRMPD) spectroscopy in conjunction with quantum chemical calculations. The obtained spectra of [4Ta,C,2H]^+^ reveal a dominance of vibrational bands of a H_2_Ta_4_C^+^ carbide dihydride structure over those indicative for a HTa_4_CH^+^ carbyne hydride one, as is unambiguously verified by studies employing various methane isotopologues. Because methane dehydrogenation by metal cations M^+^ typically leads to the formation of either MCH_2_^+^ carbene or HMCH^+^ carbyne hydride structures, the observation of a H_2_MC^+^ carbide dihydride structure implies that it is imperative to consider this often‐neglected class of carbonaceous intermediates in the reaction of metals with hydrocarbons.

## Graphical abstract

Tantalum carbide dihydride cations are identified as products in the reaction of methane with gas‐phase Ta_4_^+^ clusters by IR spectroscopy, in conjunction with density‐functional theory calculations.

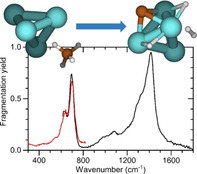

Activation of the C−H bond in small hydrocarbons, like methane, attracts currently considerable research efforts because of its potential utilization in industrial processes employed for the production of liquid fuels and other valuable chemical commodities, such as methanol and higher hydrocarbons. Such chemical processes often require harsh conditions, which makes them energetically and thus commercially costly. At the same time, these conditions hamper the elucidation of the detailed reaction mechanism and a molecular level understanding of the chemistry involved in the activation processes. However, such understanding is imperative for the rational design of state‐of‐the‐art catalysts. In this regard, single‐atom catalysts and small clusters have proven potent in research endeavors aiming at uncovering the microscopic mechanisms of elementary catalytic reactions.^[1]^

Activation of the C−H bond in methane at mild conditions has been found to be feasible in reactions with third‐row transition metal cations in a variety of environments,^[2]^ in line with further studies performed in flow tubes^[3]^ and using guided‐ion‐beam techniques.^[1f]^ Among these metals, tantalum has been identified as a favorable element with the prospect of serving as a successful catalyst, particularly in light of the experimental observation of the catalytic non‐oxidative coupling of methane facilitated by silica‐supported tantalum hydrides under realistic conditions.^[4]^ Furthermore, cationic tantalum oxides were identified to exhibit a peculiar reactivity (as for example a possible formation of CH_2_O and CH_3_OH in the reaction with CH_4_),^[5]^ and a tantalum atomic cation induces coupling of methane and carbon dioxide.^[6]^ The activity towards methane is not only limited to single Ta atom compounds. Ta_8_O_2_^+^ clusters, for example, enable the non‐oxidative methane coupling in the gas phase,^[7]^ and bare Ta_4_^+^ clusters exhibit remarkable reaction properties in methane dehydrogenation.^[8]^ For the latter, this concerns in particular the potential structure of the reaction products and their reactivity towards the formation of value‐added product in subsequent reactions.

Among the pertinent results from recent investigations on this topic (i.e.Ta_4_^+^),^[8]^ we note: (i) The reaction of Ta_4_^+^ with methane starts with the formation of [4Ta,C,2H]^+^ (here and in the following we denote the species determined through the mass‐spectrometric experiments in a form that gives only the material's stoichiometry), followed by a facile second dehydrogenation reaction. (ii) First‐principles density functional theory (DFT) calculations predicted that in the first step of the reaction, a H_2_Ta_4_C^+^ carbide dihydride structure is energetically favored over carbene (Ta_4_CH_2_^+^).^[8]^ To the best of our knowledge, prior to that study^[8]^ only carbenes (‐CH_2_) and carbyne hydrides (‐H and ‐CH) have been identified as primary reaction products. For example, infrared multiple photon dissociation (IRMPD) spectroscopy showed that methane dehydrogenation mediated by atomic transition metal cations usually results in carbene structures, including TaCH_2_^+^ for the Ta^+^ ion.^[9]^ (iii) Interestingly, the [4Ta,C,2H]^+^ species was also found to be very reactive towards O_2_, yielding value‐added products attributed to syngas and/or formaldehyde with a selectivity of >50 %.^[10]^

Given these findings it is imperative to know the exact structure of the [4Ta,C,2H]^+^ product. For this, we employ the combination of IRMPD spectroscopy and first‐principles quantum calculations. This combination of experimental and theoretical methodologies has emerged in the past two decades as a powerful tool for the elucidation of the structures of mass‐selected metal ions and clusters.[^1f^, ^11^] The methods have been employed previously to investigate tantalum‐based compounds, in particular bare tantalum cluster cations^[12]^ and cationic cluster oxides.^[13]^ Whereas investigations of atomic cations and their methane dehydrogenation products for different elements[^9^, ^14^] are rather abundant, including studies of relevant reaction products from other precursor molecules,^[15]^ only a few studies have addressed the IRMPD spectra of products of methane activation by metal clusters. These studies include the characterization of intermediates from the entrance channel (i.e. methane adsorption and insertion of the metal into the C−H bond) for cationic gold^[16]^ and platinum^[17]^ clusters.

The IRMPD spectroscopic experiment has been performed in a molecular beam instrument coupled to the intracavity free‐electron laser (FELICE).^[18]^ The low energies of IR photons require the absorption of several tens of them in order to break strong covalent bonds. Due to the Ta−C and Ta−H bond energies (see Supporting Information), the high photon densities within the laser cavity are advantageous for the IR structural characterization of products formed upon reacting tantalum clusters with methane. For this, Ta_4_^+^ clusters were formed using laser ablation, and reacted with various isotopical forms of methane in a flow‐tube type reaction channel. The spectral range probed is 290–1800 cm^−1^, fully covering the characteristic vibrational modes of all potential [4Ta,C,2H]^+^ products, with the exception of the symmetric and antisymmetric C−H stretching modes of the carbene close to 3000 cm^−1^. The spectra were recorded with an optimized overlap of the cluster beam with that of the free‐electron laser to obtain a compromise between the signal‐to‐noise ratio and the spectral resolution.

The information pertaining to the different species has been obtained by theoretical explorations of the atomic arrangements, electronic structure, and vibrational characteristics (see Table S2) using Born‐Oppenheimer spin‐density‐functional theory molecular‐dynamics (BO‐SDFT‐MD) calculations.^[19]^ In addition, IR spectra and reaction energetics were modeled with DFT calculations at the PBE/TZVP level of theory,^[20]^ with Grimme's D2 dispersion correction^[21]^ as implemented in the Gaussian package.^[22]^ In light of the excellent agreement between both computational methods (shown in Tables S1 and S2), the experimental measurements are compared here to results from the latter one. Harmonic frequencies were scaled by a factor of 0.96 to correct for anharmonicities, and convoluted with a 20 cm^−1^ (47 cm^−1^ FWHM) Gaussian line‐shape function to facilitate comparison to the experiment. Details of the experiment and the respective computations and further results, including additional spectra, are presented in the SI.

Figure 1 compiles the experimental results for [4Ta,C,2H]^+^ (Panel A) and selected isotopologues (Panels B and C) in comparison with the calculated vibrational spectra of three different isomeric structures: (I) a carbide dihydride–H_2_Ta_4_C^+^, (II) a carbyne hydride–HTa_4_CH^+^, and (III) a carbene–Ta_4_CH_2_^+^. The IRMPD spectra of [4Ta,C,2H]^+^, [4Ta,^13^C,2H]^+^, and [4Ta,C,H,D]^+^ displayed in Figure 1 were all obtained from fragmentation into the [4Ta,C]^+^ mass channel.

**Figure 1:**
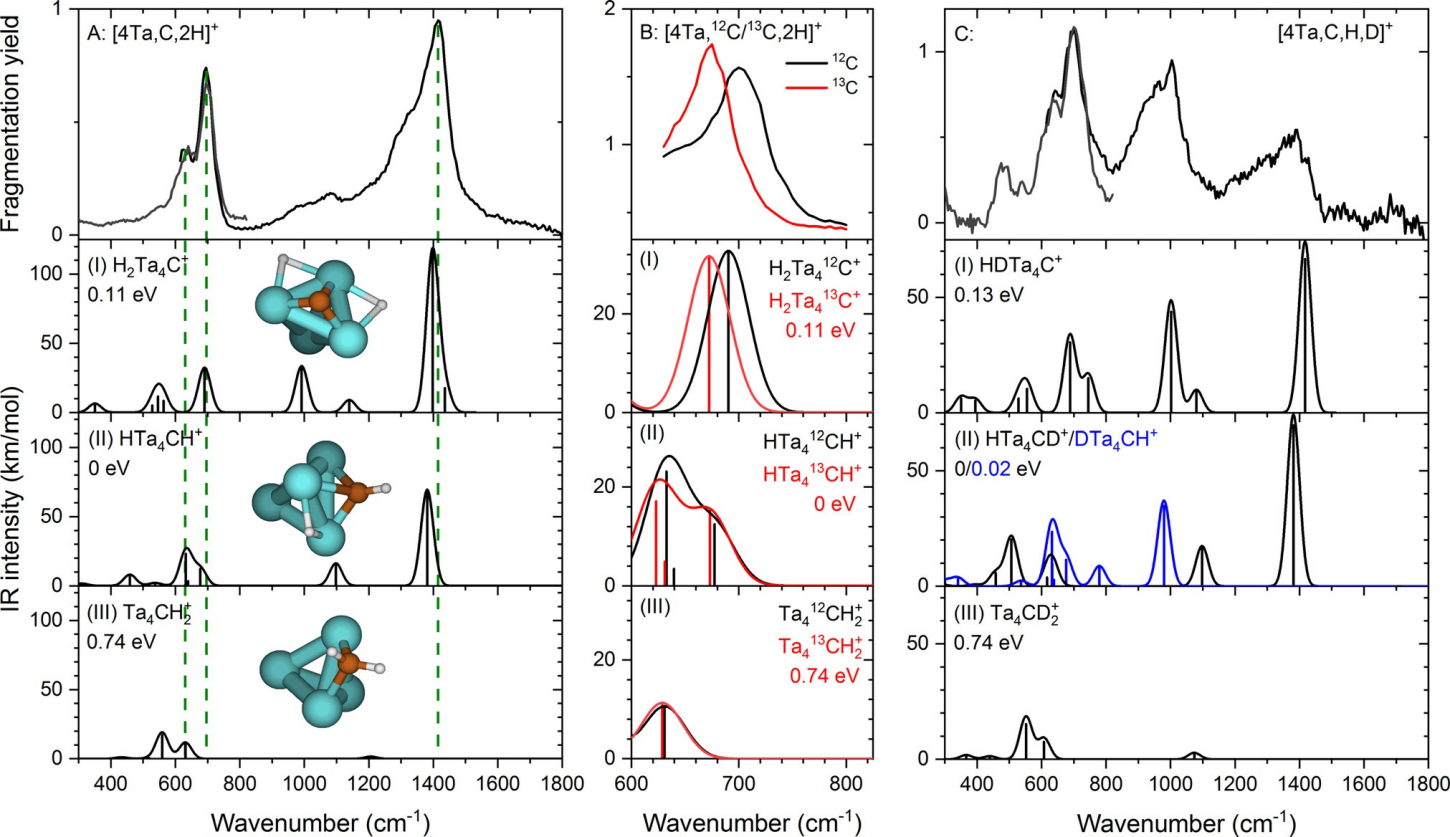
Experimental IRMPD spectra (top row) for (A) [4Ta,C,2H]^+^, and isotopologues (B) [4Ta,^12^C/^13^C,2H]^+^, and (C) [4Ta,C,H,D]^+^_._ Calculated (scaled) harmonic spectra at the PBE+D2/TZVP level of theory, corresponding to the species in A–C, computed for three different structures (see inserts in the leftmost column), that is: (I) a carbide dihydride, (II) a carbyne hydride, and (III) a carbene, are shown in rows 2, 3, and 4, respectively. The energy values (in units of eV) in the various panels correspond to the relative energies of the corresponding structural isomers relative to the lowest one (marked as the zero of the relative energy scale).

The experimental IRMPD spectrum of [4Ta,C,2H]^+^ exhibits two main bands peaking at 695 and 1400 cm^−1^. Both bands are asymmetric, suggesting the presence of other resonances, with the pronounced shoulder at 630 cm^−1^ being the clearest indication. Between 900 and 1200 cm^−1^ another, weaker, absorption is observed. Comparing this spectrum to the calculated spectra of different structural isomers of [4Ta,C,2H]^+^, we observe first the lack of any significant IR activity for the Ta_4_CH_2_^+^ carbene structure (Figure 1 A‐III) near the prominent peak at 1400 cm^−1^_._ The two other isomers under consideration, the carbide dihydride H_2_Ta_4_C^+^ and the carbyne hydride HTa_4_CH^+^, offer a better correspondence with the observed spectral characteristics. In both cases, the most intense band is predicted to originate from a Ta‐H stretch vibration, while bands with considerable intensity are expected from a Ta‐C stretch vibration, as well as other Ta‐H stretching modes. Isotopic substitution provides further insights regarding the origin of the different vibrational bands, since the consequently observed spectral band shifts are directly correlated with the involvement of the substituted atom in the vibrational displacements. Thus, upon ^13^C substitution, the 695 cm^−1^ band red‐shifts by ≈27 cm^−1^ (Panel B), while its position remains unaffected upon deuteration (Panel C), confirming involvement of the carbon and not of the hydrogen. The observed ^13^C red‐shift is very similar to that predicted (26 cm^−1^) for the Ta‐C stretch vibration of the carbide dihydride structure. For the carbyne hydride species, bands involving motion of the C−H group are predicted at similar albeit slightly lower frequencies, but their shift upon ^13^C substitution is less pronounced. It thus appears plausible that the 695 cm^−1^ experimental band is due to the carbide dihydride, and the 630 cm^−1^ due to the carbyne hydride.

Whereas the band at 695 cm^−1^ (including the 630 cm^−1^ shoulder) stays relatively unaffected by a single H/D substitution ([4Ta,C,H,D]^+^, Figure 1 C), the relative intensity of the 1400 cm^−1^ band gets significantly reduced, and pronounced new spectral features appear. The most prominent new feature is centered around 1000 cm^−1^, and weaker ones at 480 and 540 cm^−1^ are observed, too. These observations can again be rationalized by the presence of both the carbyne hydride and the carbide dihydride structures, in line with the very close energies calculated by us for these two species. The change in the relative intensities of the monodeuterated species with regard to the perprotio (i.e. isotopically unsubstituted) one agrees well with the trend found in the calculations. Furthermore, bands originating from Ta‐D stretch vibrations are predicted to occur around 1000 cm^−1^, but an interpretation of the spectra of the monodeuterated compound is less straightforward than those of the other isotopologues. One reason for this is the necessary inclusion of two isotopomers of the carbyne hydride, as different positions of the hydrogen and deuterium atoms must be considered.

The IRMPD spectrum for [4Ta,C,2D]^+^ could not be recorded directly, as its mass channel is strongly contaminated by IR induced fragmentation from the [4Ta,C,4D]^+^ adsorption product, which is much more abundant due to the earlier reported large kinetic isotope effect in the dehydrogenation of methane.^[8]^ However, we were able to record the IRMPD spectrum of [4Ta,C,2D]^+^ complexed with methane. Spectra recorded using both CD_4_ and CH_2_D_2_ as reactant are displayed in Figure 2 (A,B). While this “self‐tagging” facilitates the dissociation process due to a relatively low binding energy of the adsorbed methane molecule, it also brings about an increased number of bands in the spectrum. In both sets of experimental spectra in Figure 2, the higher frequency range is dominated by bands of the tagging molecule. This is evident from a) the high similarity between the spectra of [4Ta,C,2D]^+^⋅L (L=CD_4_ or CH_2_D_2_) and the spectra of the tagged bare cluster [4Ta]^+^⋅L, and b) the correlation to the frequencies reported for free deuterated methane (CD_4_ or CH_2_D_2_, respectively),^[23]^ indicated by the blue dashed lines in Figure 2 (A,B). Thus, the region above 1000 cm^−1^ cannot be used as a diagnostic for the structure of the [4Ta,C,2D]^+^ core. However, a pronounced split band with maxima at 690 and 720 cm^−1^ is clearly unrelated to the tagging molecule, and we interpret this band as due to [4Ta,C,2D]^+^ itself. This doublet, which is observed in both spectra and is not dependent on the degree of deuteration of the precursor molecule, appears to agree most with the carbide dihydride system, for which a doublet associated to Ta‐C stretch and Ta‐D‐Ta bending vibrations, is predicted to occur at 687 and 706 cm^−1^, respectively (see Figure 2 (I)). Interestingly, the spectrum lacks any direct evidence for the presence of the carbyne hydride. This appears in contradiction with the spectra for the [4Ta,C,2H]^+^ and [4Ta,C,H,D]^+^, but may be attributed to result from the tagging process. Furthermore, no bands are observed in the region below 1000 cm^−1^, even though the calculations predict their presence. Their absence is likely due to their low calculated IR intensity, preventing an efficient excitation to energies exceeding the fragmentation threshold. Such effects have been described in the literature.^[24]^ However, the absence of further bands should not be taken to imply the sole presence of the carbide dihydride, particularly since the absent bands associated with this species are of a similar calculated IR (low) strength as those absent for the carbide dihydride.

**Figure 2:**
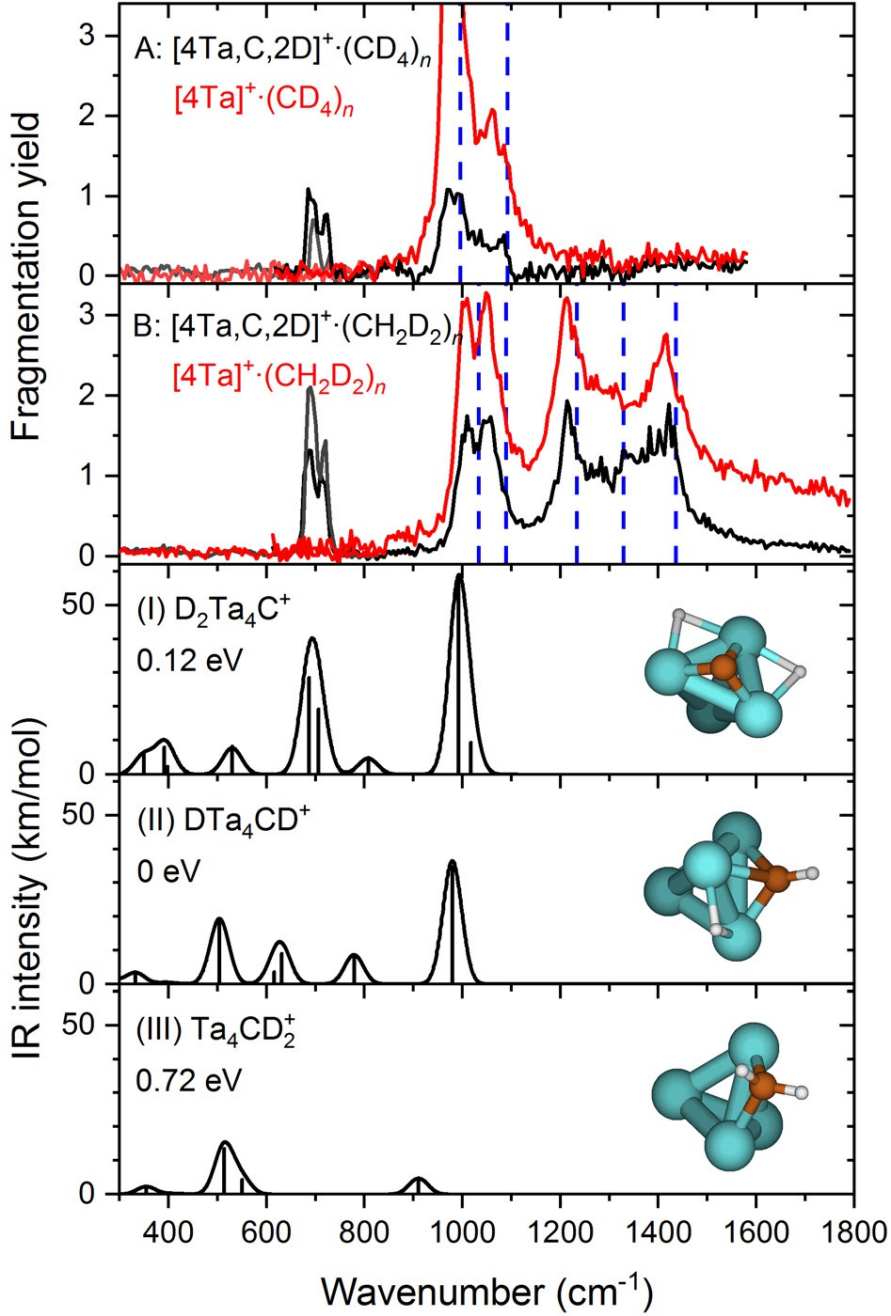
Experimental IRMPD spectra (black curves) of [4Ta,C,2D]^+^⋅methane, with methane isotopologues CD_4_ (panel A) and CH_2_D_2_ (panel B). Panels (I–III): calculated spectra for three different [4Ta,C,2D]^+^ structures: H_2_Ta_4_C^+^ carbide dihydride (I), HTa_4_CH^+^ carbyne hydride (II), and Ta_4_CH_2_^+^ carbene (III), modelled at the PBE+D2/TZVP level of theory. Vertical dashed lines represent the experimental IR frequencies for the corresponding free methane isotopologues.^[23]^ The energy values (in units of eV) in the various panels correspond to the relative energies of the corresponding structural isomers relative to the lowest one (marked as the zero of the relative energy scale).

Alternatively, it could be argued that the experiments may include a certain bias towards the carbyne hydride and carbide dihydride systems in general, because of the significantly lower relative intensities of the carbene system above 800 cm^−1^. However, if the carbene species would have been the dominant population, its significant presence should have been readily visible in the 500–700 cm^−1^ spectral range. Instead, the observation that only the carbide dihydride exhibits the above‐noted doublet in the spectra of the tagged clusters (compare Figure 2 (I) with Figure 2 (II)), lends support to our conclusion that this species is the main product of the reaction of Ta_4_^+^ with methane.

In summary, IRMPD spectra of [4Ta,C,2H]^+^, an intermediate of the Ta_4_^+^‐mediated methane dehydrogenation, recorded in the 290–1800 cm^−1^ spectral range, exhibit three strong absorption features at 630, 695, and 1400 cm^−1^. Based on observed shifts upon various isotopic substitutions and quantum chemical calculations, these modes can be conclusively attributed to the Ta‐H and Ta‐C vibrations. While all spectra exclude an assignment to a carbene species, a discrimination between the carbyne hydride and carbide dihydride is less straightforward. Nevertheless, theoretical analysis of the measured spectra, and in particular the ones corresponding to the perdeuterated compounds, enables an unambiguous identification of the carbide dihydride structure (i.e. H_2_Ta_4_C^+^) dominating over a carbyne hydride one (i.e. HTa_4_CH^+^). Carbide dihydrides, as the one uncovered here, represent a class of carbonaceous species, which were typically regarded as energetically unfavorable over other structures.[^14b^, ^15a^] However, the present study shows that this assumption may not hold in general and consequently probing for the presence of carbide dihydrides should be included in investigations of reaction intermediates in heterogeneously catalyzed reactions of metals with hydrocarbons.

## Conflict of interest

The authors declare no conflict of interest.

## Supporting information

As a service to our authors and readers, this journal provides supporting information supplied by the authors. Such materials are peer reviewed and may be re‐organized for online delivery, but are not copy‐edited or typeset. Technical support issues arising from supporting information (other than missing files) should be addressed to the authors.

Supplementary
