# Supplementary material for: Carbide Dihydrides: Carbonaceous Species Identified in Ta4 +‐Mediated Methane Dehydrogenation
Source: Angew Chem Int Ed Engl. 2020 Oct 22;59(52):23631–5. doi: 10.1002/anie.202010794 (PMC7814672; doi:10.1002/anie.202010794)
Supplement: Supplementary file 1 — Supplementary [file ANIE-59-23631-s001.pdf]

Angewandte Chemie
Eine Zeitschrift der Gesellschaft Deutscher Chemiker

Supporting Information

Carbide Dihydrides: Carbonaceous Species Identified in Ta_4^+-Mediated Methane Dehydrogenation

*Jozef Lengyel, Nikita Levin, Frank J. Wensink, Olga V. Lushchikova, Robert N. Barnett, Uzi Landman, Ueli Heiz, Joost M. Bakker, and Martin Tschurl**

anie_202010794_sm_miscellaneous_information.pdf

I. EXPERIMENTAL DETAILS

The molecular beam apparatus, which is connected to the beamline of FELICE, is described in detail elsewhere.[1] Tantalum clusters were generated by laser ablation of a rotating and translating tantalum rod, irradiated by the focused 2nd harmonic of a Nd:YAG laser in the presence of a pulse of helium (General Valve Series 9, 8 bar stagnation pressure) that acts both as promotor of cluster formation and as carrier gas. The formed clusters were entrained by the helium pulse into a flow tube, which was filled with the respective isotopologue of pure methane, introduced by a second solenoid valve at a reduced stagnation pressure of typically a few 100 mbar. The clusters and their reaction products were subsequently expanded into the vacuum, forming a molecular beam. The formed beam was skimmed and subsequently shaped by a 8 x 0.45 mm horizontal slit aperture before entering the interaction zone where it was irradiated by the IR laser. Directly after irradiation, all ions were orthogonally extracted into a reflectron time-of-flight mass spectrometer with resolution ($m/\Delta m$) of better than 1500. Mass spectra were recorded using a chevron-style multi-channel plate detector and a digitizer (Acqiris DP310). The experiment was operated at twice the frequency of the IR laser (5 Hz), allowing to record reference mass spectra.

The intracavity IR laser FELICE produced IR radiation in ~8 microsecond long macropulses consisting of picosecond near-transform limited micropulses, with a spectral width (full-width at half-maximum) of ca. 0.6% of the central frequency. FELICE was operated with a repetition rate of 5 Hz in a wavelength range from about 300 cm^{-1} to 1800 cm^{-1} using two different electron energies. The optical beam is characterized by a 55 mm Rayleigh range, and spectra were recorded at 300 mm from the FELICE focus. The pulse energy, inferred from outcoupling a fraction of the radiation, ranged from ~0.6 J at 300 cm^{-1} to 0.5 J at 1800 cm^{-1} with a maximum of 1.1 J at 600 cm^{-1}, translating to a maximum IR fluence of 5 J/cm^2 at 1800 cm^{-1}. The wavelength was calibrated using a grating spectrometer.

IRMPD spectra are recorded by monitoring the fragmentation reaction $[4Ta,C,2H]^+ \rightarrow Ta_4C^+ + H_2$, which is endothermic by 1.2 eV for the carbene, 1.8 eV for the carbide dihydride, and 1.9 eV for the carbyne hydride (calculated at PBE+D2/TZVP level of theory) and requires the absorption of multiple photons to induce dissociation. The spectra are presented as the fragmentation yield $Y_f(\omega)$, defined as

$$Y_f(\omega) = \ln\left(1 + \frac{I(\omega)_F - I_F}{I(\omega)_P}\right) \qquad \text{(Eq. S1)}$$

with $I(\omega)_P$ and $I(\omega)_F$ the integrated intensities of the precursor and fragment (product) ions after IR laser irradiation at frequency ω, and I_F the integrated intensity of the fragment ion without laser irradiation. The fragmentation yield was then corrected with the laser power. As shown in Figure S2, this approach minimizes the influence of fluctuation of the ion intensity and gives spectra with higher signal-to-noise ratio than log-depletion spectra, for which the remaining population in the parent mass channel is monitored as a function of frequency: $Y_d(\omega) = \ln\left(1 + \frac{I(\omega)_P}{I_P}\right)$.

The $^{12}C/^{13}C$ isotopic labeling spectra (Figure 1B) were performed by filling the flow tube with a 50:50 mixture of $^{12}CH_4$ and $^{13}CH_4$, and by simultaneously recording the IRMPD spectrum for both species, thereby eliminating potential calibration errors.

IRMPD spectra for complexes $P^+ \cdot M$ of an ion P^+ (P = [4Ta,C,2D] and Ta_4) with methane M, based on depletion suffer from ingrowth by fragmentation of $P^+ \cdot M_n$ (n=2,3,..). To eliminate this, the branching ratio B of all $P^+ \cdot M_n$ complexation products to all P^+ products is calculated

$$B = \frac{\sum_{n=1\dots} I_{P^+M_n}}{\sum_{n=0\dots} I_{P^+M_n}} \qquad \text{(Eq. S2)}$$

The depletion yield is then calculated as

$$Y_d(\omega) = -\ln\left(\frac{B(\nu)}{B_0}\right) \qquad \text{(Eq. S3)}$$

This methids is valid as long as irradiation of each $P^+ \cdot M_n$ leads to elimination of M and the formation of $P^+ \cdot M_{n-1}$. We carefully verified that each $P^+ \cdot M_n$ exhibited the same spectral response, suggesting each M is merely a spectator.

II. CALCULATIONS

Our experimental observations were supported with quantum chemistry calculations using both a commercially available DFT calculation at the PBE+D2/TZVP level of theory in the Gaussian package as well as the Born-Oppenheimer spin-density-functional theory molecular-dynamics (BO-SDFT-MD) method[2] that we used before in our investigation of Ta_4^+ reactivity.[3]

Calculations using the Gaussian 09 package

The calculations were performed for the three different Ta structures: the carbide dihydride, the carbyne hydride, and the carbene to identify their vibration modes. We used our previously observed energetic minima from the BO-SDFT-MD calculations as the initial structures[3] and

equilibrated them in molecular dynamics runs to find the most stable isomers. Molecular dynamics were run on the BLYP/def2SVP potential energy surface, nuclei were propagated according to classical equations of motion; the constant temperature of 298 K was maintained with Nosé-Hoover thermostat. The total length of the simulations was 1 ps, with a time step of ~1 fs (i.e. 1000 steps). From the trajectory, final structure was taken and re-optimized at the PBE/TZVP level of theory with Grimme's D2 dispersion correction.[4] All calculations were performed with the Gaussian 09 software package,[5] ABIN code was used for molecular dynamics.[6]

Calculations using the BO-SDFT-MD method

The theoretical explorations of the atomic arrangements, electronic structure, and vibrational characteristics of the tantalum clusters and their derivatives were also performed with the use of the Born-Oppenheimer spin density-functional theory molecular dynamics (BO-SDFT-MD) method[2] with norm-conserving soft (scalar relativistic for Ta) pseudopotentials[7] and the generalized gradient approximation (GGA) with the PBE functional for electronic exchange and correlations.[4a] A detailed description of the method is found in our previous article.[3]

In these calculations we have used a plane-wave basis with a kinetic energy cut-off E_c = 62 Ry, which yields convergence. This corresponds to a real-space grid spacing of 0.4 a_0 (Bohr radius); the real-space grid spacing for the density (and potential) was 0.133 a_0 corresponding to E_c = 555 Ry. In the construction of the Ta pseudopotentials the valence electrons, $5d^3$ and $6s^2$, were characterized by core radii r_c(s) = 2.55 a_0 and r_c(d) = 2.00 a_0, with the *s* orbital treated as local, and (unoccupied) $6p^0$ core radius r_c(p) = 3.00 a_0. Additionally for Ta there is a non-linear core correction with the pseudo-core containing 10% of the [Xe]$4f^{14}$ core. For the carbon atom pseudopotential the valence $2s^2$ and $2p^2$ electrons were treated with r_c(s) = r_c(p) = 1.45 a_0, with the *p* orbital treated as local. The 1s electron of the H atoms was described by local pseudopotential with r_c(s) = 0.95 a_0.

The energy minimization to find the optimal cluster geometry was done with a steepest-descent method. The convergence criteria were that the maximum force magnitude on any particle is less than 0.0005 Hartree/Bohr and that the average over all particles is less than 0.00025 Hartree/Bohr. In some cases BO-SDFT-MD simulations of typically a few picosecond duration at 300 K (that is, canonical, constant temperature, simulations, with stochastic thermalization) were used to ensure that the resulting optimal configurations were stable; a time-step of 0.25 fs was used in these simulations.

In light of the excellent agreement of both methods, PBE+D2/TZVP and BO-SDFT-MD (shown in Tables S1 and S2), the experimental measurements are compared in the text of the manuscript, only to results from the former.

III. EXPERIMENTAL RESULTS

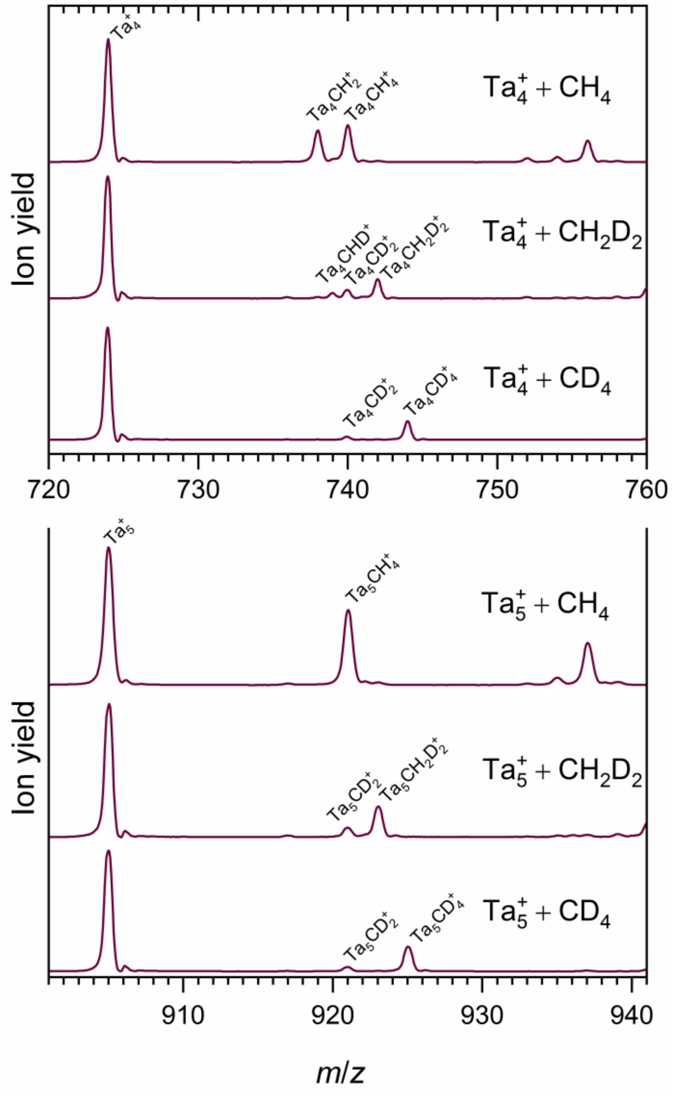

Figure S1. Mass spectra of Ta_4^+ clusters exposed to three different methane isotopologues. The observed products can be explained by the comparison with results from studies in ion traps[3] and indicate that similar reaction conditions were present in both experiments. First, products of dehydrogenation are only observed for Ta_4^+, while for Ta_5^+ only loosely bound methane species created by the adiabatic expansion result. Second, the reactions with CH_2D_2 clearly show that a large isotopic effect (KIE) results in the dehydrogenation of the molecule, as the main product of this reaction is $Ta_4CD_2^+$. The KIE, however, also results in a significantly reduced dehydrogenation efficiency toward CD_4, whose product must also be considered to be isobaric with species comprising on O-atom. The same isobaric interference similarly explains the existence of the peak being labeled by $Ta_5CD_2^+$, but instead represents the monoxide of Ta_5^+.

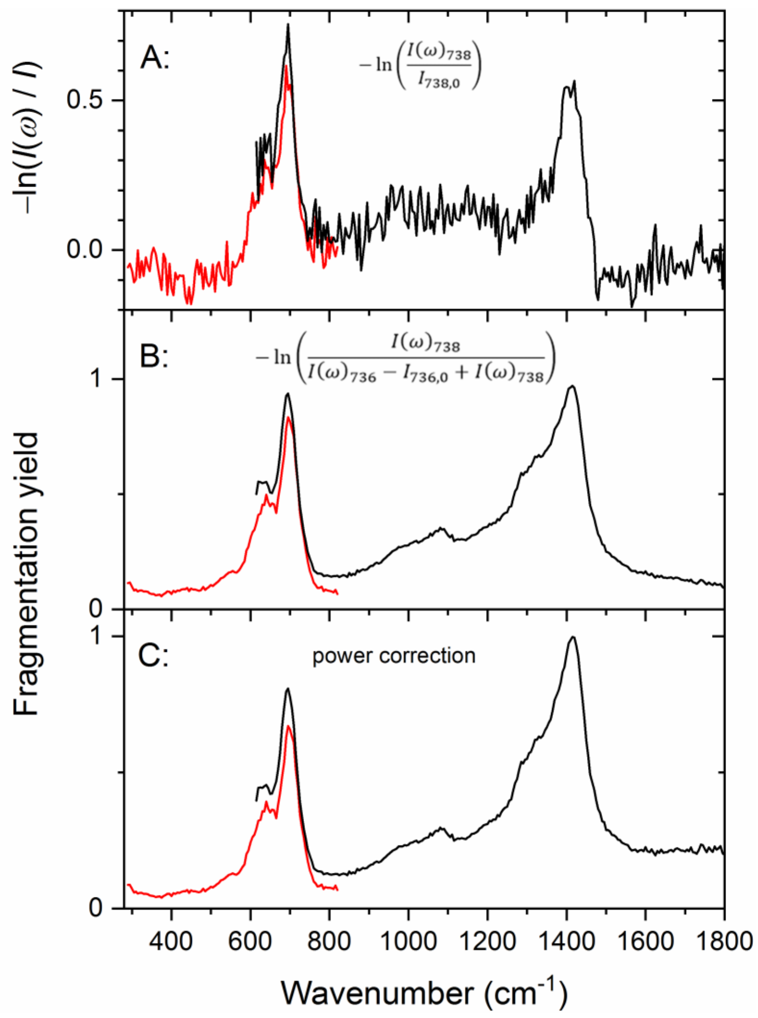

Figure S2. Experimental IRMPD spectra of $[4Ta,C,2H]^+$ illustrated as a log-depletion spectrum (A) and by the fragmentation yield (B) defined by Eq. S1. The fragmentation yield is further normalized to the macropulse energy (C).

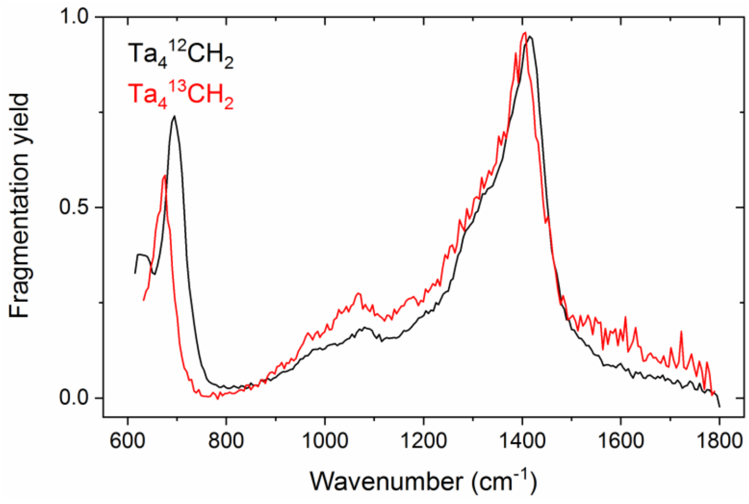

Figure S3. IRMPD spectra of [4Ta,^{12}C,2H]$^{+}$ and [4Ta,^{13}C,2H]$^{+}$ recorded in separate measurements. While the majority of features only exhibit slight deviations in the two spectra, which are attributed to the uncertainties in the measurements, the band structure between 600 and 800 cm^{-1} is clearly shifted. A more quantifiable comparison is provided in simultaneous measurements shown in Figure 1B.

IV. VIBRATIONAL FREQUENCES

Table S1. Calculated vibrational frequencies (in cm^{-1}) of $[4Ta,C,2H]^+$ for three different structural motifs: the carbide dihydride $H_2Ta_4C^+$, the carbyne hydride HTa_4CH^+, and the carbene $Ta_4CH_2^+$ using PBE/TZVP level of theory, with Grimme's D2 dispersion correction. The present harmonic vibrational frequencies are not scaled. Note that the carbyne hydride consists of two isomers with no equivalent hydrogens; for [C,H,D] isotopologes the upper value represents DTa_4CH^+ structure and the lower value represents HTa_4CD^+ structure.

H_2TaC^+	C,2H	^{13}C,2H	C,2D	C,H,D	$HTaCH^+$	C,2H	^{13}C,2H	C,2D	C,H,D	$TaCH_2^+$	C,2H	^{13}C,2H	C,2D	C,H,D
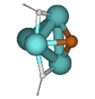	1496	1496	1060	1476	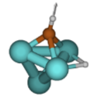	2993	2984	2204	2993<br>2204	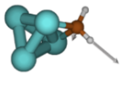	2944	2933	2182	2907
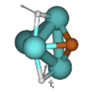	1456	1456	1033	1043	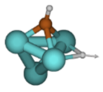	1439	1439	1020	1020<br>1439	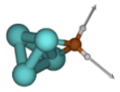	2865	2860	2071	2124
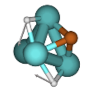	1187	1187	842	1125	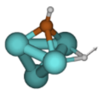	1143	1143	811	812<br>1143	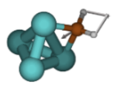	1256	1250	948	1118
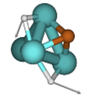	1033	1033	735	776	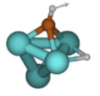	706	694	657	704<br>659	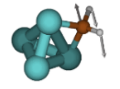	657	648	573	633
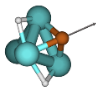	719	693	715	717	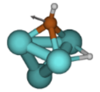	666	651	642	665<br>642	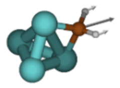	582	563	535	575
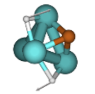	587	586	414	578	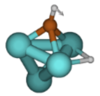	659	642	525	659<br>528	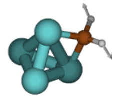	568	568	405	497
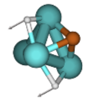	569	568	408	411	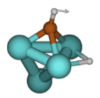	559	552	413	559<br>410	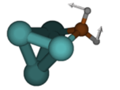	540	535	426	457
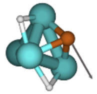	550	531	552	551	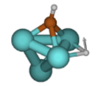	477	477	346	354<br>477	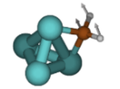	451	441	369	382
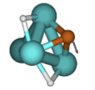	365	352	364	365	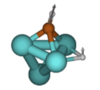	322	312	294	313<br>298					

Table S2. Calculated vibrational frequencies (in cm^{-1}) of $[4Ta,C,2H]^+$ for three different structural motifs: the carbide hydride $H_2Ta_4C^+$, the carbyne hydride HTa_4CH^+, and the carbene $Ta_4CH_2^+$ using the Born-Oppenheimer spin-density-functional-theory molecular dynamics (BO-SDFT-MD) method,[2] employing generalized exchange-correlation approximation, with the PBE functional.[4a]

H_2TaC^+	C,2H	^{13}C,2H	C,2D	C,H,D	$HTaCH^+$	C,2H	^{13}C,2H	C,2D	C,H,D	$TaCH_2^+$	C,2H	^{13}C,2H	C,2D	C,H,D
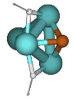	1498	1498	1056	1481<br>1037	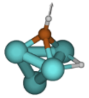	2998	2989	2205	2998<br>2205	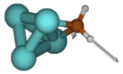	2951	2941	2185	2911<br>2126
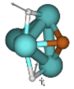	1454	1454	1026		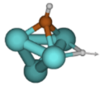	1449	1449	1027	1027<br>1449	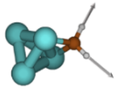	2867	2862	2071	
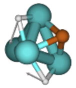	1166	1166	824	1130<br>776	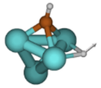	1118	1118	793	739<br>1118	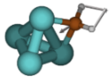	1264	1259	952	1126
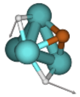	1055	1055	750		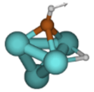	690	675	653	689<br>654	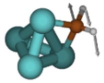	662	654	569	633
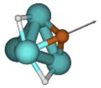	711	685	705	710	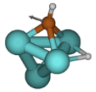	658	641	633	657<br>634	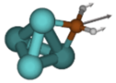	575	560	530	569
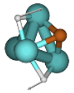	596	595	409	596<br>386	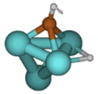	648	637	508	648<br>512	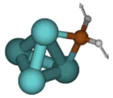	561	557	395	460
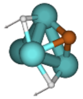	565	545	385		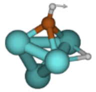	540	534	401	540<br>456	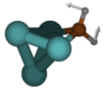	546	543	428	488
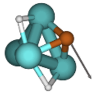	541	541	562	565	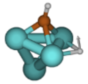	458	457	334	345<br>396	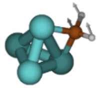	452	441	374	382
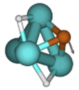	342	330	337	341	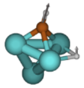	304	295	276	292<br>282					

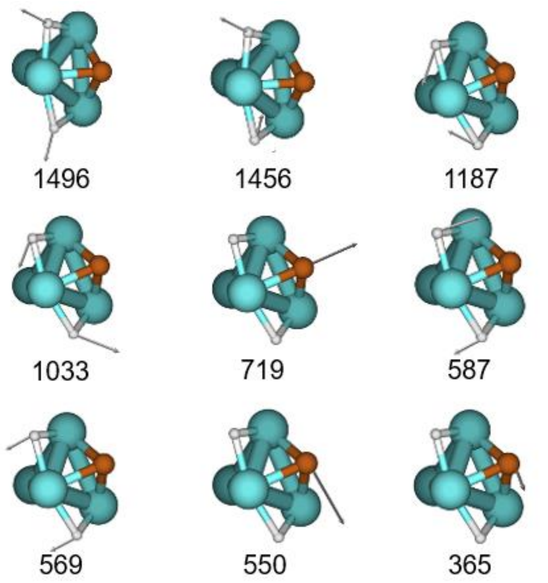

Figure S4. Vibrational modes and the respective frequencies in cm^{-1} calculated for the carbide dihydride structure at the PBE+D2/TZVP level of theory.

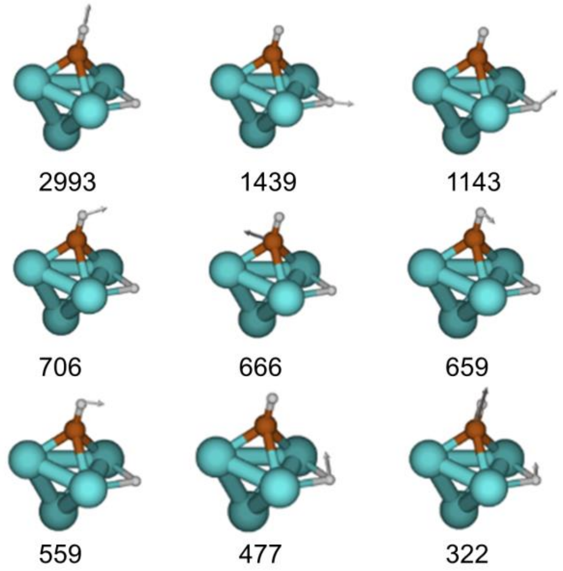

Figure S5. Vibrational modes and the respective frequencies in cm^{-1} calculated for the carbyne hydride structure at the PBE+D2/TZVP level of theory.

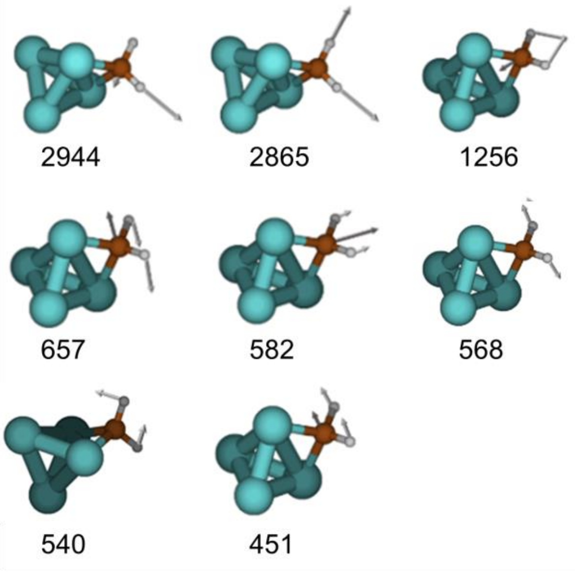

Figure S6. Vibrational modes and the respective frequencies in cm^{-1} calculated for the carbene structure at the PBE+D2/TZVP level of theory.

V. BENCHMARK CALCULATIONS

Table S3 summarizes benchmark calculations of enthalpies of stepwise clustering of tantalum cation compared to experimental values.[8] The results of the PBE functional lie much closer to the experimental values in comparison with other functionals, e.g. B3LYP or TPSSh. Looking at different basis sets, the results of PBE+D2/TZVP methods is in reasonable agreement with experiment (to within 0.1 eV). Table S4 compares the calculated bond energies for Ta^+–H and Ta^+–CH_x, x = 0–3, using multiple levels of theory, with the experimental values reported by Armentrout and co-workers. PBE functional perform much better for Ta^+–H bonds than the other two functionals. In contrast, all the Ta^+–C chemical bond energies calculated with the use of the B3LYP functional yield better agreement with the experiment, whereas calculations that employ the PBE functional seem to systematically overestimate the calculated bond energies compare to the experimental values. Nevertheless, all three DFT functionals predict the same trends of bond energies.

Table S3. Enthalpies of Ta clustering calculated at various levels of theory (all in eV) compared to the experimental results. Enthalpies are calculated at 298.15 K within the harmonic approximation.

Property	PBE/ def2TZVP	PBE/ LANL2DZ	PBE/ SDD	B3LYP/ def2TZVP	B3LYP/ LANL2DZ	TPSSh/ def2TZVP	TPSSh/ LANL2DZ	Exp.[8]
$\Delta H(Ta^+ + Ta \rightarrow Ta_2^+)$	–6.82	–7.08	–6.72	–6.27	–5.98	–6.87	–7.54	–6.90
$\Delta H(Ta_2^+ + Ta \rightarrow Ta_3^+)$	–6.76	–6.73	–6.71	–6.13	–7.02	–7.07	–7.16	–6.67
$\Delta H(Ta_3^+ + Ta \rightarrow Ta_4^+)$	–7.64	–7.56	–7.43	–7.09	–7.15	–8.08	–8.14	–7.71

Table S4. Comparison of calculated and experimental bond energies (in eV) for Ta^+–H and Ta^+–CH_x, x = 0–3. Experimental values are adopted from Ref. [9].

Species	PBE/ def2TZVP	PBE/ LANL2DZ	PBE/ SDD	B3LYP/ def2TZVP	B3LYP/ LANL2DZ	TPSSh/ def2TZVP	TPSSh/ LANL2DZ	Exp.[9]
Ta^+–H	2.45	2.47	2.44	2.00	1.99	1.93	2.30	2.39±0.08[10]
Ta^+–CH_3	3.50	3.77	3.50	3.13	3.39	3.13	3.37	2.69±0.14[11]
Ta^+–CH_2	5.14	5.31	4.95	4.68	4.86	4.57	4.68	4.81±0.03[11]
Ta^+–CH	6.35	6.71	6.30	5.69	6.03	5.61	5.95	5.82±0.16[11]
Ta^+–C	5.01	4.99	4.66	4.34	3.42	4.33	3.17	3.79±0.04[12]

VII. CARTESIAN COORDINATES OF THE OPTIMIZED STRUCTURES

Note that structures (coordinates in Å) are optimized at the PBE+D2/TZVP level of theory.

Ta4+HH_C (carbide dihydride)

Ta	0.888366	-0.510143	-1.275735
Ta	0.910180	-0.508680	1.307649
Ta	2.927362	0.386982	0.105590
Ta	0.259000	1.612494	0.127118
C	1.840612	1.517150	1.326784
H	2.835650	-1.056469	1.292781
H	-0.760347	0.594585	1.321126

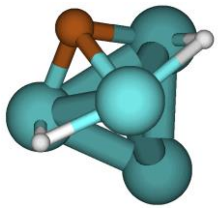

Ta4+H_CH (carbyne hydride)

Ta	0.902234	-0.431636	-1.247175
Ta	0.952178	-0.482341	1.311114
Ta	2.972336	0.415910	-0.046702
Ta	0.105948	1.571467	0.103930
C	1.877056	1.684355	1.080540
H	2.191459	2.313873	1.932110
H	-0.734170	0.511137	1.407870

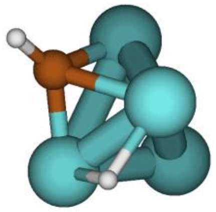

Ta4+CH2 (carbene)

Ta	0.916290	-0.449906	-1.334759
Ta	0.916290	-0.449906	1.334757
Ta	2.883362	0.446828	0.000000
Ta	0.281124	1.616579	-0.000001
C	2.218122	2.446526	0.000000
H	2.471390	3.009693	0.924462
H	2.471391	3.009695	-0.924461

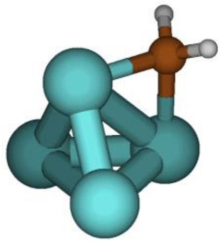

REFERENCES

[1] a) J. M. Bakker, V. J. F. Lapoutre, B. Redlich, J. Oomens, B. G. Sartakov, A. Fielicke, G. von Helden, G. Meijer, A. F. G. van der Meer, *J Chem Phys* **2010**, *132*, 074305; b) M. Haertelt, V. J. F. Lapoutre, J. M. Bakker, B. Redlich, D. J. Harding, A. Fielicke, G. Meijer, *J Phys Chem Lett* **2011**, *2*, 1720-1724.

[2] R. N. Barnett, U. Landman, *Phys Rev B* **1993**, *48*, 2081-2097.

[3] J. F. Eckhard, T. Masubuchi, M. Tschurl, R. N. Barnett, U. Landman, U. Heiz, *J Phys Chem C* **2018**, *122*, 25628-25637.

[4] a) J. P. Perdew, K. Burke, M. Ernzerhof, *Phys Rev Lett* **1996**, *77*, 3865-3868; b) S. Grimme, *J Comput Chem* **2006**, *27*, 1787-1799.

[5] M. J. Frisch, G. W. Trucks, H. B. Schlegel, G. E. Scuseria, M. A. Robb, J. R. Cheeseman, G. Scalmani, V. Barone, G. A. Petersson, H. Nakatsuji, X. Li, M. Caricato, A. V. Marenich, J. Bloino, B. G. Janesko, R. Gomperts, B. Mennucci, H. P. Hratchian, J. V. Ortiz, A. F. Izmaylov, J. L. Sonnenberg, D. Williams-Young, F. Ding, F. Lipparini, F. Egidi, J. Goings, B. Peng, A. Petrone, T. Henderson, D. Ranasinghe, V. G. Zakrzewski, J. Gao, N. Rega, G. Zheng, W. Liang, M. Hada, M. Ehara, K. Toyota, R. Fukuda, J. Hasegawa, M. Ishida, T. Nakajima, Y. Honda, O. Kitao, H. Nakai, T. Vreven, K. Throssell, J. A. Montgomery, J. E. Peralta, F. Ogliaro, M. J. Bearpark, J. J. Heyd, E. N. Brothers, K. N. Kudin, V. N. Staroverov, T. A. Keith, R. Kobayashi, J. Normand, K. Raghavachari, A. P. Rendell, J. C. Burant, S. S. Iyengar, J. Tomasi, M. Cossi, J. M. Millam, M. Klene, C. Adamo, R. Cammi, J. W. Ochterski, R. L. Martin, K. Morokuma, O. Farkas, J. B. Foresman, D. J. Fox, Gaussian 09, Revision D.01, Wallingford CT, **2009**.

[6] D. Hollas, O. Svoboda, M. Ončák, P. Slavíček, ABIN, Version 1.0, https://github.com/PHOTOX/ABIN, **2015**.

[7] N. Troullier, J. L. Martins, *Phys Rev B* **1991**, *43*, 1993-2006.

[8] P. B. Armentrout, B. L. Kickel, in *Organometallic Ion Chemistry, Vol. 15* (Ed.: B. S. Freiser), Springer, Dordrecht, **1996**, pp. 1-45.

[9] P. B. Armentrout, *Chem Eur J* **2017**, *23*, 10-18.

[10] X. G. Zhang, C. Rue, S. Y. Shin, P. B. Armentrout, *J Chem Phys* **2002**, *116*, 5574-5583.

[11] L. G. Parke, C. S. Hinton, P. B. Armentrout, *J Phys Chem C* **2007**, *111*, 17773-17787.

[12] C. S. Hinton, F. X. Li, P. B. Armentrout, *Int J Mass Spectrom* **2009**, *280*, 226-234.
